# Comparison of Functional Outcome of Total and Unicompartmental Knee Arthroplasty Using Computer-Assisted Patient-Specific Templating

**DOI:** 10.1155/2021/5524713

**Published:** 2021-06-24

**Authors:** Atef Mohamed Morsy, Emad Gaber Elbana, Ahmed Gaber Mostafa, Mark Ashraf Edward, Mahmoud A. Hafez

**Affiliations:** ^1^The Orthopaedic Department, Beni-Suef University, Beni-Suef, Egypt; ^2^The Orthopaedic Department, Faculty of Medicine, October 6 University, Giza, Egypt

## Abstract

**Background:**

Knee arthroplasty surgeries are in ever-increasing demand. With unicompartmental knee arthroplasty (UKA), patients may benefit from a higher range of flexion and a better Knee Society Score (KSS).

**Aim:**

In this study, we compared the short-term clinical outcomes of total knee arthroplasty (TKA) and UKA using the patient-specific templating (PST) technique.

**Methods:**

Two groups of 20 knees each were divided into UKA and TKA groups depending on the Oxford criteria of UKA. Only patients with medial compartmental osteoarthritis were included. KSS, functional knee score (FKS), and ROF were assessed preoperatively and at 6 months postoperatively.

**Results:**

The TKA group has shown a significant improvement compared to the UKA group in KSS (MD = 39.35 vs. 31.2, respectively, *p*=0.003). Both TKA and UKA have shown no significant difference concerning both the FKS (MD = 32 and 31.75, respectively, *p*=0.926) and ROF (MD = 10.25 and 7.25, respectively, *p*=0.072). *Discussion*. The higher improvement of KSS in the TKA group can be attributed to the fact that patients in the TKA had significantly worse KSS preoperatively. Also, the small improvement in ROF in the UKA group might be related to their wider preoperative ROF.

**Conclusion:**

Preoperatively, the TKA group had lower KSS and ROF compared to UKA. The improvement of KSS from preoperative to postoperative was more significant in TKA. However, the TKA group has shown less range of flexion postoperatively.

## 1. Introduction

Unicompartmental osteoarthritis (OA) targets mainly the medial compartment rather than the lateral aspect of the patellofemoral joint. Almost half of these patients are suitable for unicompartmental knee arthroplasty (UKA) [1]. The current operative treatment modalities of advanced osteoarthritis are total knee arthroplasty (TKA) and unicompartmental knee arthroplasty (UKA). However, replacing all the affected and nonaffected surfaces is considered the gold standard procedure [2, 3]. On the other hand, many surgeons argue the superiority of UKA in unicompartmental cases to spare the undamaged compartments, despite having higher revision rates when compared to TKA [2, 4].

Patient-specific instrumentation (PSI) used in UKA or TKA can decrease the intraoperative time drastically, which consequently would reduce the intraoperative and postoperative complications. This is possible through the preoperative use of a CT scan or MRI to determine the size of the implant [5, 6]. There are many logistic difficulties to use PSI in Egypt; consequently, we developed a hospital-based PSI protocol under the name of patient-specific templating (PST). This protocol requires CT imaging, a dedicated engineer to design the implants, and a small 3D printer. Although previous studies have investigated the effectiveness of PSI [7], comparing the effectiveness of this technique in both UKA and TKA was not performed before.

In this study, we aim to record the short-term outcomes of TKA and UKA using PST in terms of the Knee Society Score (KSS) [8, 9], functional knee score (FKS) [8, 9], and the range of flexion (ROF).

## 2. Methods

**Figure 1:**
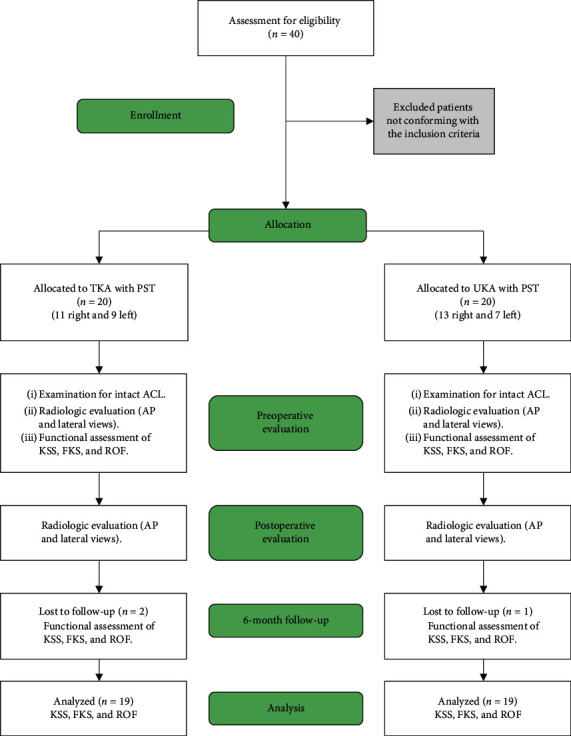
Flow diagram showing the recruitment process in the study. TKA, total knee arthroplasty; UKA, unicompartmental knee arthroplasty; PST, patient-specific templating; AP, anteroposterior; KSS, knee society score; FKS, functional score of knee society score; ROF, range of flexion.

We performed anon-randomized controlled trial on a total of 40 patients, all of whom received patient-specific templating (PST) TKA or UKA. Patients were assigned into two groups of equal sizes (20 participants in each group). Patients of group 1 received TKA, while those of group 2 received UKA. The process of patients' enrollment in the study is shown in Figure 1. The basic patients' characteristics and demographics are given in Table 1.

**Figure 2:**
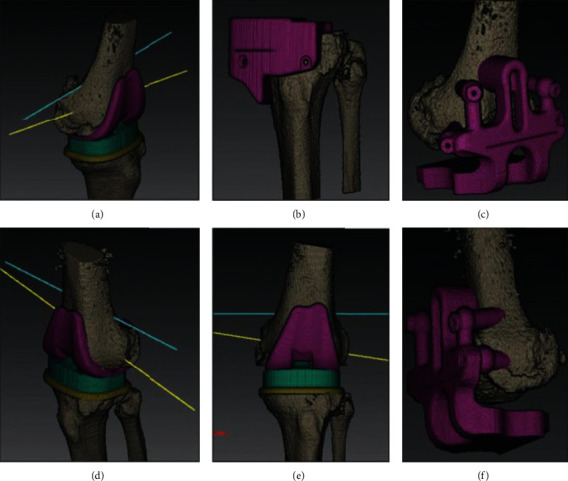
TKA planning showing the sizing, alignment, and implant positioning that matches the patient's anatomy.

**Figure 3:**
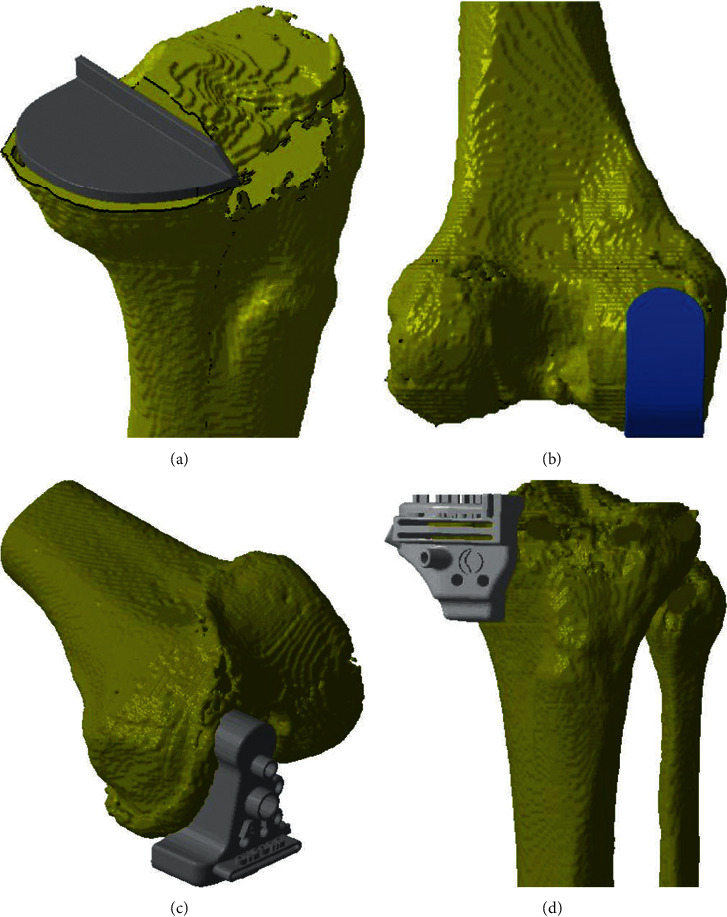
UKA planning showing the sizing, alignment, and implant positioning that matches the patient's anatomy.

**Figure 4:**
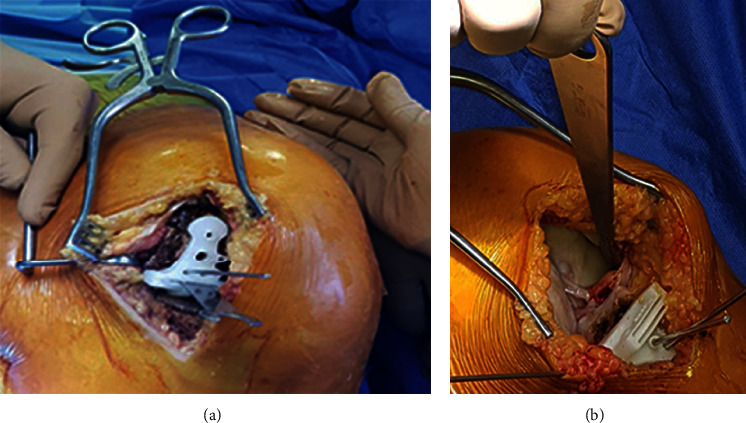
The femoral (a) and tibial (b) components of the UKA patient-specific cutting guides.

Our PST is based on commercially available patient-specific instrumentation (PSI) but with some modifications. We use CT imaging to measure the size of the required implant and to print it using hospital-based 3D printers. Figures 2 and 3 show how templating was carried out.  Figure 4 shows the UKA template after the implantation to facilitate the cutting guidance. Ethical approval was obtained from the ethical committee of the participating hospital. All patients consented to the surgical procedures and to participate in the study. We included adult patients with ages ranging between 40 and 75 of both sexes. Patients were asked about instability, underwent clinical examination for laxity and intact anterior cruciate ligament (ACL), and investigated with anteroposterior and lateral view knee X-ray to check for intact ACL, probable subluxation, and stage of the osteoarthritic bone erosion. Patients with a history of meniscal or ligamental tear, previous knee surgery, infected knee, severe varus deformities of more than 10 degrees, neurological impairment (e.g., Charcot's joint), or tricompartmental osteoarthritis were excluded. Revision cases were not included in our study. These inclusions/exclusions apply to both UKA and TKA groups. Patients' allocation into the TKA or UKA group depended on the Oxford criteria for UKA that included medial bone on bone, full-thickness lateral cartilage, intact ACL on lateral view X-ray, normal MCL on valgus stress view, and accepted patella-femoral joint in skyline X-ray. If the patient met the criteria, UKA was advised and performed. Otherwise, TKA was performed instead [10, 11].

**Figure 5:**
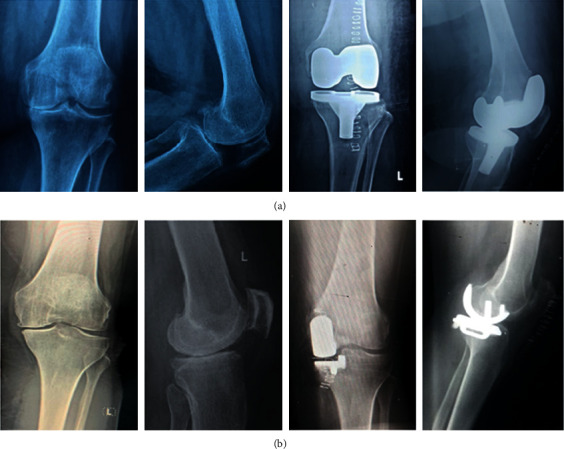
Preoperative and postoperative radiological assessment of two left knees of two female patients who undergone TKA (a) and UKA (b). Both patients had perfect operations with no complications.

In UKA and TKA, we used the Oxford partial knee system and the Vanguard knee system developed by Biomet, respectively. Details of the preoperative and postoperative assessment were documented. The clinical assessment included assessing the Knee Society Score (KSS) [8, 9], functional knee score (FKS) [8, 9], and the range of flexion (ROF). We also conducted a radiological assessment using a plain X-ray in the anteroposterior and lateral views preoperatively and postoperatively (Figure 5). Postoperative evaluation included KSS, FKS, ROF, and anteroposterior and lateral views knee X-ray. This study is not designed to examine the survival of the implants. Short-term follow-up was conducted at six months postoperatively.

Student's *t*-test and Pearson Chi-square parametric tests were used to highlight the differences between the two groups' demographic data. Data regarding age, sex, body mass index, degree, and side of varus deformity were obtained and entered using Microsoft Excel 2010. Statistical analyses were performed using IBM SPSS version 20. A *p* value of <0.05 was considered statistically significant.

## 3. Results

We included 40 patients in our study and distributed them into two groups of equal sizes. The mean duration of the follow-up was 7.4 months, ranging from 2 to 12 months. Two patients from the TKA group and one from the UKA group failed to follow-up at 6 months, but they were contacted by phone and reported any complications. Accordingly, the results of the comparison between the preoperative and postoperative values were based on 18 and 19 patients in the TKA and UKA groups, respectively.

Our study population has shown no significant difference between both groups concerning the age (64.5 vs. 61.3, respectively, *p*=0.223), female sex (12 vs. 11, respectively, *p*=0.102), or BMI (30.04 vs. 29.03, respectively, *p*=1.146) (Table 1). Preoperatively, the TKA group had statistically significant lower KSS (46.35 vs. 54.10, respectively, *p*=0.001) and lower ROF (97 vs. 107, respectively, *p*=0.001) compared to UKA. Postoperatively, we have found that the absolute ROF in the UKA group was higher than the TKA group (122.25 vs. 107.25, respectively, *p*=0.001). However, the change in ROF was higher in the TKA group with statistically insignificant results (10.25 vs. 7.25, respectively, *p*=0.075). On the other hand, there was no statistically significant difference between the two groups postoperatively regarding the KSS and the FKS (Table 2).

We noticed that the TKA group has shown better results than the UKA group regarding the KSS (mean diff. = 39.35 and 31.2, respectively, *p*=0.003). However, there was no significant difference between both groups concerning FKS (mean diff. = 32 and 31.75, respectively, *p*=0.926) and ROF (mean diff. = 10.25 and 7.25, respectively, *p*=0.072) (Table 3).

## 4. Discussion

In our study, we investigated the clinical differences in 40 patients undergoing medial UKA or TKA using PST. We have found a significant improvement in KSS in the TKA group over the UKA group. Nevertheless, when comparing the preoperative data, we have found that the UKA group had better values than the TKA group in both KSS and ROF. Both findings may be related to the significant improvement of KSS with TKA and the insignificant improvement of ROF in UKA. Unfortunately, adjusting for group differences before the initiation of this study was not feasible as the surgeons were compelled to adhere to the Oxford criteria for UKA [10, 11].

Similarly, in a systematic review of cohort studies, Kleeblad et al. agreed that range of motion (ROM) was higher in medial UKA patients compared to TKA patients, while both groups had nearly the same overall function outcome scores [12]. On the other hand, Blevins et al. conducted a retrospective analysis of 150 medial UKAs using the MAKO® robotic interactive orthopedic arm (MAKO® Surgical Corp.) and 150 TKAs using either conventional or patient-specific cutting blocks. They found that the UKA group had less postoperative numeric pain rating scale (NPRS) score, higher KSS and FKSS, faster return to work, and higher mean Forgotten Joint Score (FJS). But a drawback to this study is that both comparative groups received neither the same technique nor the same instrumentation [13]. It is also worth mentioning that using different tibial trays in UKA has an impact on implant positioning. This was investigated by Escudier et al., and they found that using a morphometric tibial tray is better than using a symmetric tibial tray in terms of decreasing the rate of overhand and improving the short-term clinical outcomes at one-year follow-up [14].

Another study published in 2017 found that UKA patients older than 75 years had less operative time and a shorter hospital stay. The initial recovery postoperatively was also better in the UKA group [15]. On the other hand, when comparing the annual revision rates of UKA and TKA, Kleeblad et al. in their systematic review found that UKA patients have a higher possibility of revision surgery [12]. In specific patients groups like severely obese patients, medial UKA was found to be more effective than TKA in improving functional knee scores and maintaining range of motion [16].

This study provides an insight into the possible outcomes of PST in UKA and TKA. It has shown that both operations provide great benefits to any patient with advanced medial compartmental osteoarthritis, regardless of age, sex, and preoperative assessment results. Nevertheless, some limitations may hinder the significance of this study. Our sample size is relatively small in comparison to other studies investigating arthroplasty; however, PST is not favored in many situations due to many logistic difficulties. Also, no studies are comparing PSI used in UKA and TKA. Another limitation is the short follow-up duration. This study was designed to examine only short-term follow-up because reviewing the survival is outside the scope of this study. Moreover, finding more matching groups preoperatively, especially in ROM, KSS, FKS, and FJS, will help in deciding which operation provides better outcomes. In our case, we used the Oxford criteria for UKA [10, 11]. However, it must be pointed out that the indications of using which technique for a patient are different according to the patient's condition and the surgeon's preference. It might also be of value in future research to compare PSI in UKA to conventional instrumentation, not only from the outcome point of view but according to the surgical techniques, learning curve, and cost-effectiveness. Whenever possible, this comparison can expand to include TKA as well. Finally, assessing other outcomes such as the cost and complications will be beneficial as well.

## 5. Conclusion

In this manuscript, we aimed at providing an insight into the short-term clinical outcomes of UKA and TKA in patients with medial compartmental osteoarthritis. Preoperatively, the TKA group had lower KSS and ROF compared to UKA. The improvement of KSS from preoperative to postoperative was more significant in TKA. However, the TKA group has shown less range of flexion postoperatively. Both UKA and TKA appear to be good treatment options for medial compartment osteoarthritis. In our study, the differences between the functional outcome of UKA and TKA were not remarkable. This was because of the sedentary lifestyle of most of our patients. Another reason is there is no significant difference between the mean age of both TKA and UKA groups (65.5 and 63.95, respectively). Accordingly, we cannot generalize and state that unicompartmental provides better functionality because studies on conventional UKA did not fully support this claim either.

## Figures and Tables

**Table 1:** Basic characteristics of the participants.

		Group 1 (TKA)	Group 2 (UKA)	*p* value
		*N* = 20	*N* = 20
Age (years)	Mean ± SD	65.5 ± 6.00	63.95 ± 9.36	0.537
	Range	56–74	48–75	

Sex	Female	12 (60%)	11 (55%)	0.749
	Male	8 (40%)	9 (45%)	

BMI (kg/m^2^)	Mean ± SD	30.04 ± 3.26	29.03 ± 2.18	0.259
	Range	24.1–35.6	25.4–32.7	

Side	Right	13 (65%)	11 (55%)	0.519
	Left	7 (35%)	9 (45%)	

Degree of varus deformity	Mean ± SD	7.5 ± 2.56	7.5 ± 2.56	1.000
	Range	5–10	5–10	

Polyethylene implant size (mm)	Mean ± SD	11.50 ± 4.05	5.45 ± 1.76	0.001
	Range	8–20	3–9	

TKA, total knee arthroplasty; UKA, unicompartmental knee arthroplasty; SD, standard deviation.

**Table 2:** Comparison between TKA and UKA groups, preoperatively and postoperatively.

		Group 1 (TKA)	Group 2 (UKA)	*p* value
		*N* = 18	*N* = 19	
Preoperative assessment				
KSS	Mean ± SD	46.35 ± 7.92	54.10 ± 6.16	0.001
	Range	24–56	45–68	
FKS	Mean ± SD	42.00 ± 8.18	40.00 ± 7.43	0.423
	Range	30–55	30–55	
ROF	Mean ± SD	97.00 ± 9.38	115.00 ± 6.49	0.001
	Range	85–115	100–125	

Postoperative assessment at six months				
KSS	Mean ± SD	85.70 ± 6.28	85.30 ± 5.24	0.828
	Range	69–96	75–92	
FKS	Mean ± SD	74.00 ± 12.42	71.75 ± 10.04	0.532
	Range	55–95	55–90	
ROF	Mean ± SD	107.25 ± 8.38	122.25 ± 2.55	0.001
	Range	95–125	120–125	

TKA, total knee arthroplasty; UKA, unicompartmental knee arthroplasty; KSS, Knee Society Score; FKS, functional knee score; ROF, range of flexion; SD, standard deviation.

**Table 3:** Comparison of the difference between the postoperative and preoperative values of Knee Society Score, functional knee score, and the range of flexion in the two groups.

	Group 1 (TKA)			Group 2 (UKA)			*p* value
	*N* = 18			*N* = 19			
	Mean difference	SD	SE	Mean difference	SD	SE	
KSS	39.35	7.74	1.73	31.20	5.08	1.14	0.003
FKS	32.00	8.18	1.83	31.75	8.93	2.00	0.926
ROF	10.25	4.72	1.06	7.25	5.50	1.23	0.072

TKA, total knee arthroplasty; UKA, unicompartmental knee arthroplasty; KSS, Knee Society Score; FKSS, functional knee score; ROF, range of flexion; SD, standard deviation; SE, standard of error.

## Data Availability

The data used to support the findings of this study are available from the corresponding author upon request.

## Abbreviations

UKA: Unicompartmental knee arthroplasty
TKA: Total knee arthroplasty
PST: Patient-specific templating
KSS: Knee Society Score
FKS: Functional knee score
ROF: Range of flexion
ROM: Range of motion
ACL: Anterior cruciate ligament
MCL: Medial cruciate ligament
AP: Anteroposterior
ECAR: Egyptian Community Arthroplasty Registry
NPRS: Numeric pain rating scale
FJS: Forgotten joint scores.

## Additional Points

*Clinical Correlation.* Performing UKA instead of TKA in patients with unicompartmental knee osteoarthritis may provide slightly better joint functionality in some patients if done using PSI techniques.
